# Association between serum estradiol levels on the hCG trigger day and euploid blastocyst rate in preimplantation genetic testing cycles: a retrospective cohort study

**DOI:** 10.3389/fendo.2026.1935196

**Published:** 2026-10-14

**Authors:** Jun Shuai, Weiwei Liu, Xinyue Sun, Qi Zhang, Hong Ye, Huali Deng, Guoning Huang

**Affiliations:** 1 Center for Reproductive Medicine, Women and Children’s Hospital of Chongqing Medical University, Chongqing, China; 2 Center for Reproductive Medicine, Chongqing Health Center for Women and Children, Chongqing, China

**Keywords:** blastocyst formation rate, estradiol, euploid blastocyst rate, generalized estimating equations, preimplantation genetic testing

## Abstract

**Background:**

While serum estradiol (E2) predicts ovarian response and endometrial receptivity, its role in embryonic development remains unclear. This study assessed the association between E2 levels on the day of human chorionic gonadotropin (hCG) trigger and the euploid blastocyst rate (EBR) in preimplantation genetic testing (PGT) cycles.

**Methods:**

This retrospective study included 3,082 couples who underwent PGT between 2017 and 2022, comprising 18,036 oocytes and 5,009 blastocysts screened using next-generation sequencing. Oocytes were stratified into tertiles based on serum E2 levels on the hCG trigger day: Group 1 (<3149 pg/mL), Group 2 (3149–4725.66 pg/mL), and Group 3 (≥4725.67 pg/mL). Associations between serum E2 levels and both blastocyst formation rate (BFR) and EBR were assessed using univariate stratified analyses and multivariable generalized estimating equation (GEE) models (crude, Model I, Model II) accounting for patient-level clustering. Restricted cubic spline (RCS) analysis with three knots, collinearity diagnostics (variance inflation factor, VIF), and formal E2 × total gonadotropin (Gn) dose interaction tests were additionally performed.

**Results:**

Univariate stratified analyses showed subgroup-specific patterns: higher E2 generally correlated with lower blastocyst formation rate (BFR) yet higher EBR. In fully adjusted GEE Model II, E2 was not associated with BFR (Group3 vs Group1: aOR 1.050, 95%CI 0.910–1.212, P = 0.502; P-trend=0.653), while high E2 was associated with higher EBR (aOR 1.295, 95%CI 1.028–1.631, P = 0.028; P-trend=0.047). RCS revealed no significant overall E2–BFR (P-overall=0.107; P-nonlinearity=0.055) or E2–EBR associations (P-overall=0.100; P-nonlinearity=0.119), although the EBR curve showed an upward high-E2 trend consistent with tertile results. No multicollinearity was detected (all VIF <6, range 1.137–5.399), and no significant E2–Gn interactions existed (all P>0.05). Sensitivity analysis excluding oocyte number yielded consistent EBR estimates (aOR 1.232, 95%CI 1.006–1.508, P = 0.044).

**Conclusion:**

In PGT cycles, elevated hCG-trigger-day E2 was not associated with BFR but was modestly associated with higher EBR. These observational findings require prospective validation and should not be interpreted as evidence of a causal or protective effect of E2 on embryonic chromosomal integrity.

## Background

Preimplantation genetic testing (PGT)—comprising blastocyst culture, trophectoderm biopsy, and genetic analysis—enables the detection of chromosomal abnormalities, single-gene disorders, and structural rearrangements in embryos (1, 2). Compared with conventional *in vitro* fertilization, PGT significantly improves live birth rates and reduces the risks of miscarriage and congenital anomalies, making it a key strategy for optimizing pregnancy outcomes in patients at high risk of chromosomal or monogenic abnormalities (3–6). Although PGT is effective in identifying embryos with chromosomal abnormalities, optimization of oocyte and embryo quality during controlled ovarian stimulation (COS) to increase the likelihood of obtaining blastocysts and euploid embryos remains a major challenge. Hormone levels during ovarian stimulation are critical determinants of follicular development, oocyte competence, and subsequent embryonic growth. Serum estradiol (E2), a steroid hormone primarily secreted by granulosa cells during folliculogenesis and widely used as a biomarker in COS protocols, is central to assessing ovarian response and determining the optimal timing for human chorionic gonadotropin (hCG) administration. Although E2 is an established predictor of ovarian responsiveness and endometrial receptivity (7–9), its impact on embryonic development—particularly blastocyst formation and euploidy—remains insufficiently understood.

Elevated serum E2 levels are considered indicative of robust follicular development and may facilitate the retrieval of a greater number of competent oocytes (10). Conversely, both animal and clinical studies have suggested that abnormal E2 concentrations may alter the follicular microenvironment, impair oxidative stress homeostasis, and disrupt meiotic spindle assembly (11–14). Elevated E2 levels have been associated with reduced blastocyst formation rates (BFRs), impaired implantation potential (15), lower oocyte quality, and increased embryo aneuploidy rates (16). Conversely, several studies have reported that patients with a high E2 response exhibit significantly higher BFRs, clinical pregnancy rates, and live birth rates compared with those with a low E2 response (10, 17). These conflicting findings may be attributed to small sample sizes, heterogeneity of the study populations, and insufficient adjustment for potential confounding factors.

To date, investigations into the association between serum E2 levels on the hCG trigger day and both BFR and EBR in PGT cycles remain limited. Furthermore, few studies have simultaneously evaluated these two outcomes within a single framework, and even fewer have explored whether the effects of E2 vary across distinct PGT subgroups. The present study was designed to address these knowledge gaps by systematically examining the relationship between serum E2 levels and both BFR and EBR in a large PGT cohort. Subgroup analyses were performed according to PGT type, COS protocol, and the number of oocytes retrieved, aiming to elucidate potential heterogeneity in E2-associated effects on embryonic developmental competence and provide novel insights for optimizing ovarian stimulation strategies, enhancing embryo quality, and ultimately improving reproductive outcomes.

## Methods

### Study design and population

This retrospective, single-center observational cohort study was conducted at the Reproductive Medicine Center of Chongqing Health Center for Women and Children. De-identified data from all first PGT cycles performed between 2017 and 2022 were extracted from the institutional reproductive medicine management system. The study population comprised patients who underwent PGT for monogenic disorders, aneuploidy, and structural rearrangements (PGT-M/A/SR). Regarding PGT-SR, only cases in which one partner carried a reciprocal or Robertsonian translocation were included. Additionally, only cycles conducted using gonadotropin-releasing hormone (GnRH) agonist or GnRH antagonist protocols were considered. The exclusion criteria were as follows: missing PGT or E2 data; both partners carrying chromosomal structural abnormalities; one partner carrying both a reciprocal and a Robertsonian translocation; PGT-SR cases involving other structural abnormalities (e.g., inversions, deletions, or complex translocations, due to limited sample size and high heterogeneity); cycles involving thawed oocytes or re-thawed embryos; oocyte or sperm donation cycles; and cycles conducted with mild stimulation or high-progesterone stimulation protocols that could interfere with E2 measurement. The final cohort included 3,082 PGT cycles and 5,009 biopsied blastocysts. The institutional Ethics Committee approved this study (approval number: 2025-RGI-03), and the requirement for informed consent was waived in light of its retrospective design.

### Ovarian stimulation and laboratory protocols

Each patient underwent a personalized COS regimen, designed by experienced clinicians based on the woman’s age, ovarian reserve, body mass index (BMI), and prior response to stimulation. Detailed descriptions of both GnRH agonist and antagonist protocols have been reported previously (18). From gonadotropin stimulation days 4–5 onwards, transvaginal ultrasound and serum hormone measurements were performed regularly to monitor follicular growth. Final oocyte maturation was triggered with recombinant hCG when at least three follicles measured ≥17 mm or two follicles measured ≥18 mm in diameter. Serum E2 levels were measured on the morning of hCG administration. Oocyte retrieval was performed 36 hours later under transvaginal ultrasound guidance, followed by intracytoplasmic sperm injection 4–6 hours post-retrieval. The protocols for intracytoplasmic sperm injection and subsequent embryo culture have been described previously (19, 20). On day 5 or 6 post-oocyte retrieval, blastocysts were assessed by senior embryologists using the Gardner and Schoolcraft grading system, with those graded ≥3BC selected. Trophectoderm biopsy involved the removal of 5–10 cells from each blastocyst using laser-assisted hatching; subsequently, the specimens were analyzed using next-generation sequencing (NGS) at Yikon Genomics. Sequencing data were processed using ChromGo (Yikon Genomics) to determine chromosomal status. In NGS profiles, mosaicism was defined as intermediate copy number changes of 20–80%, whereas changes <20% and >80% were classified as euploidy and aneuploidy, respectively. Chromosomal abnormalities were defined to include both aneuploidy and mosaicism. Details of the NGS procedure have been reported elsewhere (21). BFR was calculated as the number of blastocysts graded ≥3BC divided by the number of two-pronuclei zygotes, multiplied by 100%. EBR was defined as the proportion of euploid blastocysts among all biopsied blastocysts, expressed as a percentage.

### Statistical analyses

To minimize potential bias due to subjective cut-off points and unequal group sizes, serum E2 tertile cut-offs were derived from the distribution of E2 values across the 3,082 independent cycles, and each oocyte was assigned to an E2 tertile based on its corresponding cycle-level E2 value: Group 1, E2 <3,149 pg/mL (n = 6,024 oocytes); Group 2, E2 3,149–4,725.66 pg/mL (n = 6,000); and Group 3, E2 ≥4,725.67 pg/mL (n = 6,012). Individual oocytes were selected as the unit of analysis to allow precise tracking of each oocyte’s PGT outcome, particularly blastocyst euploidy status, and to facilitate detailed statistical evaluations of BFR and EBR. Continuous variables are presented as mean ± standard deviation, while categorical variables are summarized as frequencies or percentages. For comparisons among multiple groups, one-way analysis of variance (ANOVA) was used for normally distributed data, while the Kruskal–Wallis test was applied for non-normally distributed data. Categorical variables were compared using the chi-square test.

Missing data were handled using complete-case (listwise) deletion for the primary analyses. Among the 18,036 oocytes, only AMH (n = 521, 2.89%) and AFC (n = 28, 0.16%) had missing values, while all other variables were complete. After listwise deletion, 17,487 oocytes were included in the fully adjusted Model II analyses. Multiple imputation by chained equations (MICE) was additionally performed as a sensitivity analysis, with five imputed datasets generated using predictive mean matching for continuous variables and pooled estimates obtained using Rubin’s rules.

Associations between serum E2 levels and BFR and EBR were evaluated using multivariable logistic regression models with generalized estimating equations (GEE), with each cycle specified as the clustering variable to account for non-independence of multiple oocytes within a cycle. An independent working correlation structure was specified, and robust (sandwich) standard errors were applied. GEE was selected over mixed-effects logistic regression because it estimates population-averaged (marginal) effects that directly address our research question, is robust to misspecification of the working correlation structure when robust standard errors are used, and demonstrates more stable convergence for correlated binary embryo-level data. Three sequential models were constructed: the crude model included only serum E2 level; Model I was additionally adjusted for female age, body mass index (BMI), infertility type, PGT category (PGT-A, PGT-M, and PGT-SR), anti-Müllerian hormone (AMH), and antral follicle count (AFC); Model II was further adjusted for controlled ovarian stimulation protocol, initial gonadotropin (Gn) dose, duration of Gn administration, total Gn dose (categorical variable), and number of oocytes retrieved.

Collinearity diagnostics were performed for all covariates in Model II using tolerance and variance inflation factor (VIF), with VIF < 10 considered acceptable. Pairwise collinearity between total Gn dose and serum E2—the clinically most relevant pair—was additionally assessed. Interaction tests were conducted by entering E2 × total Gn dose interaction terms into Model II to evaluate whether the E2–outcome association differed across Gn dose strata (interaction P < 0.05 considered significant). All subgroup and stratified analyses were planned and exploratory, intended to assess the consistency of E2–BFR/EBR associations across clinical strata rather than to derive definitive conclusions. No formal multiple-testing correction was applied, as it would be overly conservative for exploratory observational analyses; findings are interpreted cautiously as hypothesis-generating, and between-subgroup differences in significance were not interpreted as evidence of effect modification.

To complement this categorical approach, restricted cubic spline (RCS) analysis with three knots placed at the 10th, 50th, and 90th percentiles of hCG-trigger-day E2 was additionally performed, with E2 modelled as a continuous variable and adjusted for all covariates in Model II. All statistical analyses were performed using IBM SPSS Statistics version 21.0 (IBM Corp., Armonk, NY, USA). RCS analyses were conducted in R (R Foundation for Statistical Computing, Vienna, Austria) using the rms package. A two-sided P-value < 0.05 was considered statistically significant.

## Results

### Baseline characteristics and COS outcomes

A total of 3,082 couples (18,036 oocytes) who met all previously defined inclusion and exclusion criteria were included in the analysis. Among these, 5,009 blastocysts were subjected to trophectoderm biopsy, followed by NGS for chromosomal assessment. **Table 1** summarizes the baseline clinical characteristics of the subjects stratified by tertiles of serum E2 levels on the day of hCG trigger. Female age and BMI gradually decreased across the three E2 groups (all P < 0.001), whereas anti-Müllerian hormone, antral follicle count, and serum E2 levels on the day of hCG trigger exhibited a progressive increase (all P < 0.001). Additionally, significant differences were observed among the groups in the distribution of infertility diagnoses (primary infertility, secondary infertility, and others), PGT categories (PGT-A/M/SR), indications for PGT-A (advanced maternal age, recurrent spontaneous abortion, and recurrent implantation failure), and genetic subtypes within PGT-SR (reciprocal and Robertsonian translocation). The three groups differed significantly in COS protocols, initial gonadotropin dose, stimulation duration, total gonadotropin dose, number of oocytes retrieved, number of metaphase II oocytes, two-pronuclei zygotes, and blastocyst yield.

**Table 1:** Baseline clinical characteristics of the study population.

Variable	Group 1 (n=6024)	Group 2 (n=6000)	Group 3 (n=6012)	*P*
Cycles	1121	1006	955	
Female age (years)	31.98 ± 4.71	30.85 ± 4.03	30.29 ± 3.91	<0.001**abc
BMI	22.23 ± 2.83	21.70 ± 2.56	21.20 ± 2.55	<0.001**abc
AMH (ng/mL)	3.41 ± 2.89	4.73 ± 3.12	5.87 ± 3.61	<0.001**abc^&^
AFC	9.48 ± 4.01	11.03 ± 3.82	11.82 ± 4.25	<0.001**abc
Basal FSH (mIU//mL)	5.70 ± 1.73	5.15 ± 1.23	5.17 ± 1.37	<0.001**ab
Basal E2 level (pg/ml)	32.67 ± 15.59	32.88 ± 14.52	33.37 ± 15.29	0.110^&^
E2 level on hCG-trigger day (pg/mL)	2105.09 ± 716.43	3914.76 ± 454.70	4975.98 ± 63.65	<0.001**abc^&^
Infertility diagnosis				<0.001**
Primary infertility	25.3% (1526/6024)	28.2% (1694/6000)	31.2% (1878/6012)	–
Secondary infertility	53.3% (3210/6024)	56.2% (3371/6000)	52.2% (3137/6012)	–
Others	21.4% (1288/6024)	15.6% (935/6000)	16.6% (997/6012)	–
PGT category				<0.001**
PGT-A	33.2% (2002/6024)	34.0% (2038/6000)	24.7% (1484/6012)	–
PGT-M	16.4% (988/6024)	15.6% (933/6000)	15.5% (934/6012)	–
PGT-SR	50.4% (3034/6024)	50.5% (3029/6000)	59.8% (3594/6012)	–
Indications for PGT-A^#^				
Advanced maternal age	55.8% (1118/2002)	38.2% (779/2038)	38.7% (574/1484)	<0.001**
RSA	35.9% (719/2002)	41.4% (843/2038)	36.3% (539/1484)	<0.001**
RIF	18.9% (379/2002)	22.5% (459/2038)	14.2% (211/1484)	<0.001**
Others	22.0% (440/2002)	19.5% (398/2038)	21.2% (314/1484)	0.151
Genetic category for PGT-SR				<0.001**
Reciprocal translocation	76.7% (2327/3034)	72.5% (2197/3029)	78.4% (2816/3594)	–
Robertsonian translocation	23.3% (707/3034)	27.5% (832/3029)	21.6% (778/3594)	–
Ovarian stimulation protocol				<0.001**
GnRH agonist protocol	43.6% (2626/6024)	72.8% (4369/6000)	81.7% (4911/6012)	–
Antagonist protocol	56.4% (3398/6024)	27.2% (1631/6000)	18.3% (1101/6012)	–
Initial gonadotropin dose (IU)	220.50 ± 62.09	187.43 ± 47.40	175.06 ± 42.51	<0.001**abc^&^
Duration of ovarian stimulation (days)	9.32 ± 1.63	10.11 ± 1.56	10.32 ± 1.42	<0.001**abc
Total gonadotropin dose (IU)	2128.62 ± 672.71	1973.46 ± 649.05	1840.58 ± 631.49	<0.001**abc^&^
Number of oocytes retrieved	12.06 ± 5.03	17.45 ± 5.66	21.76 ± 6.82	<0.001**abc
Number of MII oocyte	10.22 ± 4.35	15.04 ± 5.12	19.33 ± 6.57	<0.001**abc
Number of 2PN zygote	7.72 ± 3.58	11.46 ± 4.38	15.05 ± 5.83	<0.001**abc
Number of blastocysts	4.90 ± 2.70	6.95 ± 3.30	9.14 ± 4.81	<0.001**abc

BMI, body mass index; AMH, Anti-Müllerian Hormone; AFC, antral follicle count; FSH, Follicle-Stimulating Hormone; E2, serum estradiol; hCG, human chorionic gonadotropin; PGT, preimplantation genetic testing; PGT-A/M/SR, preimplantation genetic testing for aneuploidy, monogenic disorders, and structural rearrangements; RSA, Recurrent Spontaneous Abortion; RIF, Recurrent Implantation Failure; GnRH, Gonadotropin-Releasing Hormone; MII, Metaphase II; 2PN, Two Pronuclei; Distinct superscript letters (a, b, c) indicate significant pairwise differences among the three E2 groups (P < 0.05, Bonferroni-adjusted). Comparisons: 1 vs. 2 = a, 1 vs. 3 = b, 2 vs. 3 = c. ^#^Patients may present with multiple indications. ^&^Data were analyzed using the Kruskal–Wallis test due to non-normality.

### Comparison of BFR across different E2 groups and clinical factors

An exploratory univariate stratified analysis was conducted to assess differences in BFR across E2 tertiles and various clinical factors (**Table 2**). These subgroup analyses were intended to evaluate the consistency of the E2–BFR association across strata and to generate hypotheses for future research; between-subgroup differences were not interpreted as evidence of effect modification. Findings from subgroups with limited sample sizes should be interpreted with caution. In the intra-group analysis, BFR differed significantly among the three E2 tertiles with respect to PGT category, COS protocols, and PGT-A subgroups, specifically advanced maternal age and other factors. In Groups 1 and 2, BFR decreased progressively with increasing patient age. Notably, only Group 1 demonstrated an inverse association between longer stimulation duration, a greater number of retrieved oocytes, and reduced BFR—associations not observed in Groups 2 or 3.

**Table 2:** BFR in PGT cycles according to different parameters.

Variable	BFR						*P*
	Group 1 (n=3918)	*P*	Group 2 (n=3995)	*P*	Group 3 (n=4177)	*P*	
Age (years)		<0.001**		<0.001**		0.078	
< 35	46.1% (1261/2738)		41.6% (1336/3209)		40.0% (1437/3595)		<0.001**
35-39	43.5% (418/961)		34.6% (240/694)		39.5% (197/499)		0.001**
≥ 40	32.0% (70/219)		29.3% (27/92)		27.7% (23/83)		0.747
BMI (kg/m²)		0.463		0.154		0.389	
≤ 18.5	41.2% (113/274)		36.9% (173/469)		41.2% (258/626)		0.298
18.6-24.9	45.1% (1339/2972)		40.9% (1252/3058)		39.7% (1288/3247)		<0.001**
≥ 25	44.2% (297/672)		38.0% (178/468)		36.5% (111/304)		0.030*
Infertility diagnosis		0.315		0.243		0.810	
Primary infertility	42.6% (418/982)		40.8% (459/1126)		39.7% (518/1306)		0.375
Secondary infertility	45.4% (964/2122)		39.1% (902/2304)		39.3% (864/2196)		<0.001**
Others	45.1% (367/814)		42.8% (242/565)		40.7% (275/675)		0.240
PGT category		<0.001**		<0.001**		0.027*	
PGT-A	45.9% (591/1288)		40.1% (558/1390)		43.2% (449/1040)		0.011*
PGT-M	51.5% (322/625)		47.8% (272/569)		37.9% (253/668)		<0.001**
PGT-SR	41.7% (836/2005)		38.0% (773/2036)		38.7% (955/2469)		0.035*
Indications for PGT-A^#^							
Advanced maternal age	41.5% (309/745)	<0.001**	33.9% (185/545)	<0.001**	36.9% (144/390)	0.002**	0.020*
RSA	49.7% (236/475)	0.036*	43.2% (246/570)	0.056	46.7% (185/396)	0.070	0.106
RIF	40.5% (94/232)	0.070	41.1% (132/321)	0.684	43.0% (65/151)	0.973	0.881
Other factors	55.7% (166/298)	<0.001**	52.8% (160/303)	<0.001**	55.4% (124/224)	<0.001**	0.744
Genetic category for PGT-SR		0.226		0.244		0.040*	
Reciprocal translocation	42.4% (664/1566)		37.2% (549/1476)		37.6% (732/1945)		0.004**
Robertsonian translocation	39.2% (172/439)		40.0% (224/560)		42.6% (223/524)		0.527
Ovarian stimulation protocol		0.019*		0.034*		0.002**	
GnRH agonist protocol	42.6% (764/1793)		39.2% (1175/2999)		38.6% (1338/3467)		0.015*
Antagonist protocol	46.4% (985/2125)		43.0% (428/996)		44.9% (319/710)		0.206
AMH (ng/ml)		0.685		0.505		0.140	
< 1.2	45.5% (281/617)		42.7% (67/157)		27.8% (10/36)		0.104
≥ 1.2	44.7% (1429/3200)		40.0% (1507/3766)		39.9% (1587/3979)		<0.001**
Initial gonadotropin dose (IU)		0.966		0.145		0.372	
≤ 200IU	44.7% (739/1654)		41.0% (1060/2588)		40.1% (1270/3171)		0.007**
> 200IU	44.6% (1010/2264)		38.6% (543/1407)		38.5% (387/1006)		<0.001**
Duration of ovarian stimulation (days)		0.036*		0.140		0.495	
< 10	46.3% (1023/2210)		42.1% (542/1288)		41.2% (437/1061)		0.007**
10-12	42.9% (658/1533)		39.5% (978/2478)		39.2% (1106/2821)		0.040*
> 12	38.9% (68/175)		36.2% (83/229)		38.6% (114/295)		0.817
Total gonadotropin dose (IU)		0.068		0.437		0.175	
Low	45.2% (400/884)		41.5% (514/1240)		40.7% (766/1884)		0.068
Medium	46.8% (625/1336)		40.0% (598/1494)		40.2% (508/1264)		<0.001**
High	42.6% (724/1698)		38.9% (491/1261)		37.2% (383/1029)		0.012*
Oocyte retrieval		<0.001**		0.393		0.191	
< 6	55.2% (153/277)		60.0% (3/5)		71.4% (5/7)		0.672
6-15	43.9% (1276/2908)		41.0% (707/1724)		40.6% (300/739)		0.085
> 15	43.7% (320/733)		39.4% (893/2266)		39.4% (1352/3431)		0.087

BFR, blastocyst formation rate; BMI, body mass index; PGT, preimplantation genetic testing; PGT-A/M/SR, preimplantation genetic testing for aneuploidy, monogenic disorders, and structural rearrangements; RSA, Recurrent Spontaneous Abortion; RIF, Recurrent Implantation Failure; GnRH, Gonadotropin-Releasing Hormone; AMH, Anti-Müllerian Hormone. ^#^Patients may present with multiple indications.

* P < 0.05; ** P < 0.001.

In the inter-group analysis, significant differences in BFR across E2 tertiles were observed in multiple clinically relevant subgroups, including patients aged <35 years or 35–39 years, those with normal or high BMI, secondary infertility, all PGT categories (PGT-A/M/SR), the advanced maternal age subgroup within PGT-A, the Reciprocal translocation subgroup within PGT-SR, the GnRH agonist subgroup within COS protocols, the anti-Müllerian hormone ≥1.2 ng/mL subgroup, patients receiving initial gonadotropin doses ≤200 IU or >200 IU, those with stimulation durations <10 or 10–12 days, and those receiving medium or high total gonadotropin doses. Importantly, in most subgroups with significant differences, BFR exhibited a stepwise decrease across E2 tertiles, with higher E2 levels consistently associated with lower BFR.

### Comparison of EBR across different E2 groups and clinical factors

Consistent with the BFR subgroup analyses, these exploratory analyses were hypothesis-generating; between-subgroup differences were not interpreted as evidence of effect modification, and findings from small-sample subgroups warrant caution. In the intra-group analysis, significant differences in EBR among the three E2 groups were observed within the subgroups of PGT and PGT-SR categories. In Groups 1 and 2, EBR progressively decreased with increasing maternal age. In Groups 1 and 3, EBR declined with increasing BMI. Additionally, significant differences in EBR were noted within subgroups defined by infertility diagnosis, the “advanced maternal age” subgroup under PGT-A indications, and various oocyte retrieval subgroups. Notably, significant differences in EBR were found exclusively in Group 1 for the recurrent spontaneous abortion subgroup under PGT-A indications, different COS protocols, and subgroups stratified by initial gonadotropin doses (≤200 IU and >200 IU).

In the inter-group analysis, EBR varied significantly across E2 tertiles within the following subgroups: secondary and other types of infertility, the PGT-SR subgroup under the PGT category, the “other factors” subgroup under PGT-A indications, the GnRH agonist protocol subgroup under COS regimens, ovarian stimulation duration of 10–12 days, the high total gonadotropin dose subgroup, and the 6–15 oocyte retrieval subgroup. Notably, in most of these subgroups with statistically significant differences, EBR showed an increasing trend with higher E2 levels, in contrast to the decreasing trend observed for BFR (**Table 3**).

**Table 3:** EBR in PGT cycles according to different parameters.

Variable	EBR						*P*
	Group 1 (n=1749)	*P*	Group 2 (n=1603)	*P*	Group 3 (n=1657)	*P*	
Age (years)		<0.001**		0.003**		0.149	
< 35	44.3% (558/1261)		44.5% (594/1336)		47.2% (678/1437)		0.225
35-39	40.2% (168/418)		48.8% (117/240)		41.1% (81/197)		0.088
≥ 40	18.6% (13/70)		14.8% (4/27)		34.8% (8/23)		0.172
BMI (kg/m²)		0.042*		0.676		0.038*	
≤ 18.5	51.3% (58/113)		46.8% (81/173)		49.2% (127/258)		0.750
18.6-24.9	42.5% (569/1339)		44.6% (559/1252)		46.7% (601/1288)		0.099
≥ 25	37.7% (112/297)		42.1% (75/178)		35.1% (39/111)		0.449
Infertility diagnosis		<0.001**		0.445		<0.001**	
Primary infertility	46.4% (194/418)		47.1% (216/459)		52.7% (273/518)		0.098
Secondary infertility	38.0% (366/964)		43.8% (395/902)		45.4% (392/864)		0.003**
Others	48.8% (179/367)		43.0% (104/242)		37.1% (102/275)		0.012*
PGT category		<0.001**		<0.001**		<0.001**	
PGT-A	45.5% (269/591)		49.6% (277/558)		53.0% (238/449)		0.054
PGT-M	67.1% (216/322)		66.5% (181/272)		68.8% (174/253)		0.851
PGT-SR	30.4% (254/836)		33.2% (257/773)		37.2% (355/955)		0.009**
Indications for PGT-A^#^							
Advanced maternal age	36.2% (112/309)	<0.001**	46.5% (86/185)	0.294	43.1% (62/144)	0.004**	0.066
RSA	51.3% (121/236)	0.022*	50.4% (124/246)	0.748	54.1% (100/185)	0.710	0.744
RIF	41.5% (39/94)	0.393	48.5% (64/132)	0.761	50.8% (33/65)	0.696	0.443
Other factors	44.6% (74/166)	0.775	45.0% (72/160)	0.164	60.5% (75/124)	0.050	0.012*
Genetic category for PGT-SR		<0.001**		0.001**		<0.001**	
Reciprocal translocation	26.8% (178/664)		29.7% (163/549)		32.7% (239/732)		0.058
Robertsonian translocation	44.2% (76/172)		42.0% (94/224)		52.0% (116/223)		0.085
Ovarian stimulation protocol		0.012**		0.369		0.279	
GnRH agonist protocol	38.9% (297/764)		45.3% (532/1175)		46.9% (628/1338)		0.001**
Antagonist protocol	44.9% (442/985)		42.8% (183/428)		43.6% (139/319)		0.745
AMH (ng/ml)		0.115		0.644		0.601	
< 1.2	38.4% (108/281)		41.8% (28/67)		60.0% (6/10)		0.362
≥ 1.2	43.5% (622/1429)		44.7% (673/1507)		46.7% (741/1587)		0.206
Initial gonadotropin dose (IU)		0.033*		0.298		0.078	
≤ 200IU	45.2% (334/739)		43.7% (463/1060)		47.5% (603/1270)		0.180
> 200IU	40.1% (405/1010)		46.4% (252/543)		42.4% (164/387)		0.056
Duration of ovarian stimulation (days)		0.079		0.788		0.590	
< 10	44.1% (451/1023)		44.6% (242/542)		45.1% (197/437)		0.935
10-12	38.9% (256/658)		44.3% (433/978)		47.1% (521/1106)		0.004**
> 12	47.1% (32/68)		48.2% (40/83)		43.0% (49/114)		0.742
Total gonadotropin dose (IU)		0.243		0.259		0.795	
Low	45.0% (180/400)		45.9% (236/514)		47.0% (360/766)		0.801
Medium	43.0% (269/625)		42.0% (251/598)		45.1% (229/508)		0.577
High	40.1% (290/724)		46.4% (228/491)		46.5% (178/383)		0.037*
Oocyte retrieval		<0.001**		0.310		0.003**	
< 6	34.6% (53/153)		66.7% (2/3)		60.0% (3/5)		0.273
6-15	40.8% (520/1276)		42.7% (302/707)		50.7% (152/300)		0.008**
> 15	51.9% (166/320)		46.0% (411/893)		45.3% (612/1352)		0.100

EBR, euploid blastocyst rate; BMI, body mass index; PGT, preimplantation genetic testing; PGT-A/M/SR, preimplantation genetic testing for aneuploidy, monogenic disorders, and structural rearrangements; RSA, Recurrent Spontaneous Abortion; RIF, Recurrent Implantation Failure; GnRH, Gonadotropin-Releasing Hormone; AMH, Anti-Müllerian Hormone. ^#^Patients may present with multiple indications.

* P < 0.05; ** P < 0.001.

### Independent associations of serum E2 levels with BFR and EBR: GEE models and restricted cubic spline analyses

The crude model included only serum E2 levels on the hCG trigger day, categorized into tertiles (Group 1 [low], Group 2 [middle], and Group 3 [high]). Adjusted Model I was further adjusted for female age, body mass index (BMI), infertility type, PGT category (PGT-A, PGT-M, and PGT-SR), anti-Müllerian hormone (AMH), and antral follicle count (AFC). Adjusted Model II additionally accounted for controlled ovarian stimulation protocol, initial Gn dose, duration of Gn administration, total Gn dose (categorical variable), and number of oocytes retrieved.

For BFR (**Table 4**), in both the crude model and Adjusted Model I, higher E2 levels (Group 2 and Group 3 vs. Group 1) were significantly associated with a lower likelihood of blastocyst formation (all P < 0.001). However, after full adjustment in Model II, these associations were attenuated and became non-significant (Group 2: aOR 0.942, 95% CI 0.833–1.066, P = 0.343; Group 3: aOR 1.050, 95% CI 0.910–1.212, P = 0.502). No significant trend across E2 tertiles was observed in the fully adjusted model (P for trend = 0.653).

**Table 4:** Independent effects of E2 levels on BFR and EBR: a GEE analysis.

Variable	Crude model		Adjusted model I		Adjusted model II	
	OR (95% CI)	P	aOR (95% CI)	P	aOR (95% CI)	P
BFR						
Group 1	Reference		Reference		Reference	
Group 2	0.831 (0.747-0.925)	<0.001**	0.811 (0.726-0.905)	<0.001**	0.942 (0.833-1.066)	0.343
Group 3	0.816 (0.730-0.911)	<0.001**	0.809 (0.718-0.912)	<0.001**	1.050 (0.910-1.212)	0.502
*P* for trend	<0.001**		<0.001**		0.653	
EBR						
Group 1	Reference		Reference		Reference	
Group 2	1.100 (0.952-1.309)	0.280	1.014 (0.851-1.208)	0.878	1.047 (0.853-1.285)	0.659
Group 3	1.178 (0.990-1.401)	0.064	1.196 (0.998-1.433)	0.053	1.295 (1.028-1.631)	0.028*
*P* for trend	0.063		0.082		0.047*	

E2, serum estradiol; BFR, blastocyst formation rate; EBR, euploid blastocyst rate; OR, odds ratio; CI, confidence interval;aOR, adjusted odds ratio; The crude model included only the serum E2 level on the hCG trigger day (categorized into tertiles: Group 1 [low], Group 2 [middle], and Group 3 [high]). Adjusted model I was further adjusted for female age, body mass index (BMI), infertility type, PGT category (PGT-A, PGT-M, and PGT-SR), anti-Müllerian hormone (AMH), and antral follicle count (AFC). Adjusted model II was additionally adjusted for controlled ovarian stimulation protocol, initial Gn dose, duration of Gn administration, total Gn dose (categorical variable), and the number of oocytes retrieved. Only the results for E2 levels on the hCG trigger day are presented; the results for other covariates are not shown. *P* < 0.001.

* P < 0.05; ** P < 0.001.

For EBR (**Table 4**), no significant associations were observed in the crude model or Adjusted Model I. In the fully adjusted model (Model II), higher E2 levels (Group 3 vs. Group 1) were associated with an increased likelihood of euploid blastocyst formation (aOR 1.295, 95% CI 1.028–1.631, P = 0.028), whereas Group 2 showed no significant association (aOR 1.047, 95% CI 0.853–1.285, P = 0.659). A statistically significant trend across E2 tertiles was observed in Model II (P for trend = 0.047). Restricted cubic spline (RCS) analyses with three knots (placed at the 10th, 50th, and 90th percentiles of hCG-trigger-day serum E2) were performed to evaluate the continuous dose–response relationships between hCG-trigger-day serum E2 and both BFR and EBR, adjusting for covariates identical to fully adjusted GEE Model II. For BFR, the overall association between continuous serum E2 and BFR was not statistically significant (P-overall = 0.107). The test for non-linearity was borderline non-significant (P-for-nonlinearity = 0.055), suggesting a weak fluctuating pattern that did not reach the conventional threshold of statistical significance. For EBR, the overall association of continuous serum E2 with EBR was also not statistically significant (P-overall = 0.100). No significant non-linear relationship was detected (P-for-nonlinearity = 0.119). Although the global RCS tests were non-significant, the EBR curve visually suggested an upward trend at high E2 concentrations, which was directionally consistent with the positive tertile-based association observed in the GEE model. The RCS dose–response curves are presented in **Supplementary Figures 1** and **2**.

### Multivariable analysis of predictors for BFR and EBR based on model II GEE analysis

Beyond E2, several other covariates in the fully adjusted Model II were significantly associated with BFR and/or EBR (**Table 5**). For BFR, advanced maternal age was negatively associated with BFR (aOR 0.970, 95% CI 0.955–0.985, P < 0.001). In contrast, the GnRH antagonist protocol (vs. agonist) was positively associated with BFR (aOR 1.174, 95% CI 1.028–1.342, P = 0.018). A higher number of oocytes retrieved was negatively associated with BFR (aOR 0.983, 95% CI 0.974–0.991, P < 0.001). PGT-SR (vs. PGT-A) was negatively associated with BFR (aOR 0.791, 95% CI 0.705–0.888, P < 0.001), whereas no significant associations were observed for BMI, AMH, AFC, Gn dose-related variables, or E2 levels on the hCG trigger day.

**Table 5:** Factors associated with BFR and EBR according to E2 levels based on model II GEE analysis.

Variable	BFR (5009/12090)		EBR (2221/5009)	
	aOR (95% CI)	P	aOR (95% CI)	P
Age	0.970 (0.955-0.985)	<0.001**	0.949 (0.927–0.971)	<0.001**
BMI	1.005 (0.986–1.024)	0.609	0.980 (0.954–1.007)	0.144
Infertility diagnosis (Secondary infertility/Primary infertility)	1.028 (0.923–1.143)	0.618	0.775 (0.657–0.915)	0.003**
Infertility diagnosis (Others/Primary infertility)	1.065 (0.920–1.232)	0.402	0.757 (0.613–0.935)	0.010**
PGT category (PGT-M vs. PGT-A)	1.010 (0.876–1.166)	0.888	1.873 (1.502–2.336)	<0.001**
PGT category (PGT-SR vs. PGT-A)	0.791 (0.705–0.888)	<0.001**	0.418 (0.351–0.496)	<0.001**
AMH	0.996 (0.976–1.016)	0.665	1.010 (0.984–1.037)	0.438
AFC	1.000 (0.982-1.018)	0.973	0.994 (0.970–1.018)	0.632
Ovarian stimulation protocol (GnRH antagonist/agonist)	1.174 (1.028–1.342)	0.018*	0.994 (0.802–1.233)	0.960
Initial gonadotropin dose	0.999 (0.998–1.001)	0.453	0.999 (0.997–1.002)	0.727
Duration of ovarian stimulation	0.951 (0.904–1.001)	0.055	0.959 (0.885–1.037)	0.295
Total gonadotropin dose(middle group vs. low group)	1.106 (0.959–1.276)	0.165	1.036 (0.833–1.289)	0.751
Total gonadotropin dose(high group vs. low group)	1.091 (0.877–1.357)	0.435	1.173 (0.824–1.669)	0.378
Number of oocytes retrieved	0.983 (0.974–0.991)	<0.001**	0.994 (0.979–1.009)	0.418
E2 level on hCG-trigger day (Group2/1)	0.942 (0.833–1.066)	0.343	1.047 (0.853–1.285)	0.659
E2 level on hCG-trigger day (Group3/1)	1.050 (0.910–1.212)	0.502	1.295 (1.028–1.631)	0.028*

Data are presented as adjusted odds ratios (aOR) with 95% confidence intervals (CIs) derived from multivariable logistic regression models using generalized estimating equations (GEE) to account for clustering of multiple oocytes and embryos within patients. BFR, blastocyst formation rate; EBR, euploid blastocyst rate; E2, estradiol; BMI, body mass index; PGT-A, preimplantation genetic testing for aneuploidy; PGT-M, preimplantation genetic testing for monogenic disorders; PGT-SR, preimplantation genetic testing for structural rearrangements; AMH, anti-Müllerian hormone; AFC, antral follicle count; GnRH, gonadotropin-releasing hormone; hCG, human chorionic gonadotropin. *P < 0.05; **P < 0.001.

For EBR, advanced maternal age was negatively associated with EBR (aOR 0.949, 95% CI 0.927–0.971, P < 0.001). Compared with PGT-A, PGT-M was positively associated with EBR (aOR 1.873, 95% CI 1.502–2.336, P < 0.001), whereas PGT-SR was negatively associated with EBR (aOR 0.418, 95% CI 0.351–0.496, P < 0.001). In addition, both secondary infertility and other infertility diagnoses were negatively associated with EBR compared with primary infertility (all P < 0.05). Among the remaining ovarian stimulation-related variables (COS protocol, initial Gn dose, stimulation duration, and total Gn dose), no significant associations were observed with EBR.

### Collinearity and interaction analyses

Collinearity diagnostics were performed for all covariates included in the fully adjusted GEE Model II. The VIF values ranged from 1.137 to 5.399 (all < 6), indicating no significant multicollinearity in the final model (**Supplementary Table 1**). The highest VIF was observed for the high total Gn dose stratum (VIF = 5.399), followed by initial Gn dose (VIF = 4.924), both well within acceptable limits.

**Supplementary Table 1** presents pairwise collinearity statistics between total Gn dose and serum E2, the clinically most relevant pair. When both variables were modelled as categorical, the VIF values were 4.014 for total Gn dose and 1.899 for serum E2; when modelled as continuous, the VIF values were 9.686 for total Gn dose and 2.243 for serum E2. The continuous parameterization of total Gn dose yielded a higher VIF (9.686) and was not used in the final models, in which total Gn dose was treated as a categorical variable. Interaction tests revealed no significant interaction between serum E2 and total Gn dose for either BFR (P = 0.159 for continuous E2; P = 0.908 for categorical E2) or EBR (P = 0.701 for continuous E2; P = 0.927 for categorical E2), indicating that the association between E2 and blastocyst outcomes was consistent across Gn dose strata (**Supplementary Table 1**).

### Sensitivity analysis excluding oocyte number

To address potential over-adjustment by the number of oocytes retrieved—a marker of ovarian response that may share biological pathways with trigger-day E2—we re-ran the fully adjusted Model II excluding this variable (**Supplementary Table 2**). For BFR, the association between high E2 (Group 3 vs. Group 1) and blastocyst formation remained non-significant (aOR 1.108, 95% CI 0.973–1.261, P = 0.122), consistent with the primary analysis. For EBR, the highest E2 tertile remained significantly associated with a higher euploid blastocyst rate (aOR 1.232, 95% CI 1.006–1.508, P = 0.044), although the association was slightly attenuated compared with the primary Model II (aOR 1.295, P = 0.028). These findings indicate that the observed E2–EBR association is not solely attributable to adjustment for oocyte number.

Additionally, multiple imputation by chained equations (MICE) was performed as a sensitivity analysis for missing data (AMH: 2.89%; AFC: 0.16%), and the pooled estimates were consistent with those from the complete-case analysis.

## Discussion

This large retrospective cohort study involving 3,082 couples undergoing PGT investigated the association between serum E2 levels on the hCG trigger day and both BFR and EBR. Herein, univariate analyses based on E2 tertiles suggested that higher E2 levels were associated with a reduced BFR and an increased EBR; however, these associations were attenuated after adjustment for potential confounders. In multivariate GEE models, E2 concentrations at hCG trigger were not significantly associated with BFR, whereas patients with high E2 showed a modest but significant increase in EBR compared with those with low E2.

Current evidence regarding the impact of elevated E2 levels on embryonic chromosomal ploidy remains limited and inconsistent. Animal studies have shown that high E2 exposure markedly reduces blastocyst cell proliferation, impairs expansion, and increases apoptosis, ultimately leading to reduced implantation rates (15). Similarly, an early randomized controlled trial in humans reported that elevated E2 levels during the early stimulation phase in GnRH antagonist protocols were associated with lower pregnancy rates (22). However, as neither study directly evaluated BFR or EBR, the impact of high E2 on embryonic chromosomal status remains unclear. In contrast, several studies have reported that supraphysiologic E2 concentrations do not compromise *in vitro* fertilization outcomes and may even be associated with increased blastocyst formation, as well as higher clinical pregnancy and live birth rates (10, 23, 24). Furthermore, studies in frozen-thawed embryo transfer cycles (25, 26) and oocyte donation cycles (27) indicate that serum E2 levels during ovarian stimulation do not adversely affect oocyte quality or embryonic development. These discrepancies may reflect differences in study populations and stimulation protocols. Compared with previous research, our study is the first to directly assess the relationship between E2 levels and embryonic chromosomal ploidy in PGT cycles, showing that high E2 levels were not detrimental to EBR but were observationally associated with higher rates of euploid blastocyst formation, thereby providing preliminary observational evidence regarding embryonic chromosomal integrity under high E2 conditions. Importantly, the adjusted odds ratio for this association was modest (aOR 1.295; 95% CI 1.028–1.631), with a confidence interval that only marginally excluded the null value, indicating that the observed association should be interpreted with caution and does not establish a causal relationship. Complementary restricted cubic spline analyses treating E2 as a continuous variable showed no significant overall or nonlinear associations with either BFR (P-overall = 0.107; P-for-nonlinearity = 0.055) or EBR (P-overall = 0.100; P-for-nonlinearity = 0.119). Notably, the EBR spline curve exhibited a directional upward trend at high E2 concentrations, consistent with the positive tertile-based association, although the global test did not reach statistical significance. Accordingly, these findings should not be interpreted as evidence of a protective effect of E2 on embryonic chromosomal integrity.

Elevated E2 levels have been shown to interfere with glycogen synthesis and secretion in endometrial epithelial cells (28) and increase uterine contractility (29). In addition, high E2 concentrations have been demonstrated to induce apoptosis in endometrial glandular cells, thereby impairing endometrial receptivity (30). We previously reported that elevated E2 levels prior to progesterone transformation in hormone replacement therapy-based frozen-thawed embryo transfer cycles could compromise endometrial receptivity, leading to reduced pregnancy and live birth rates (31). However, beyond its adverse effects on the endometrium, few clinical studies have investigated whether high E2 increases the risk of embryonic chromosomal abnormalities, thereby indirectly impairing pregnancy outcomes. To our knowledge, this study is the first to utilize a large-scale PGT cohort to simultaneously examine the relationships between E2 and both BFR and EBR. Baseline comparisons among the three E2 tertiles revealed significant differences in multiple characteristics, including age, BMI, ovarian reserve, infertility type, PGT indication, and stimulation protocol, indicating that various confounders influenced the results. To address this, we performed both intra- and inter-group stratified analyses. Intra-group analyses revealed that under similar E2 levels, clinical factors such as age, BMI, infertility diagnosis, PGT category, and COS regimen were associated with BFR and EBR. Inter-group analyses allowed evaluation of BFR and EBR across different E2 levels within the same clinical subgroups. Our results showed that BFR decreased with increasing E2 levels across most clinically relevant subgroups, which may reflect differences in ovarian response and patient characteristics across E2 strata rather than a direct effect of E2. In contrast, EBR increased with higher E2 levels in multiple subgroups, consistent with the absence of a negative association between elevated E2 and chromosomal euploidy. After adjusting for confounders in the multivariate regression analyses using GEE, no significant association was observed between E2 levels on the hCG trigger day and BFR, suggesting that blastocyst formation may depend more on oocyte yield, ovarian responsiveness, and other clinical factors rather than directly on E2. In our previous study, advanced maternal age was associated with a lower BFR (18). In contrast, elevated E2 levels were associated with a higher EBR, suggesting that the adverse pregnancy outcomes observed in association with high E2 may be partly attributable to impaired endometrial receptivity and a shift in the window of implantation, rather than damage to embryonic genetic integrity. However, the underlying mechanisms remain to be elucidated and cannot be definitively established from the present observational data. Furthermore, it should be noted that EBR was calculated only among blastocysts that underwent trophectoderm biopsy, and thus represents a conditional measure restricted to embryos that reached the biopsy-eligible stage. Consequently, the EBR findings may not fully reflect the chromosomal status of all embryos generated in the cycle, and selection bias arising from differential blastocyst formation rates cannot be excluded. An important consideration that warrants explicit acknowledgment is the issue of residual confounding by ovarian response. Although our multivariable models included AMH, AFC, COS protocol, gonadotropin dose, and number of retrieved oocytes, serum E2 remains intrinsically and highly correlated with ovarian reserve and follicular development. Consequently, it remains unclear whether the observed association between elevated E2 and higher EBR reflects a true independent biological effect of E2 on embryonic chromosomal status, or whether E2 merely serves as a surrogate marker of favorable ovarian reserve, robust follicular recruitment, and greater oocyte yield. The attenuation of BFR and EBR associations after full adjustment in Model II further supports the possibility that E2 is largely a proxy for underlying ovarian responsiveness rather than a direct biological determinant of embryo euploidy. Notably, a sensitivity analysis excluding the number of oocytes retrieved from Model II yielded a consistent, albeit slightly attenuated, EBR association (aOR 1.232, 95% CI 1.006–1.508, P = 0.044), indicating that the observed E2–EBR association is not solely attributable to adjustment for oocyte yield. Furthermore, although the association between high E2 and elevated EBR reached statistical significance, this finding cannot be directly extrapolated to clinical pregnancy or live birth outcomes, as implantation, ongoing pregnancy, and live birth were not evaluated in the present study. Future studies incorporating more markers of oocyte competence, downstream clinical endpoints (implantation, ongoing pregnancy, and live birth), or causal inference methods such as instrumental variable analysis are needed to determine the clinical significance of these findings and disentangle these interrelated pathways.

In addition to the primary outcomes, our exploratory subgroup analyses revealed several notable findings. Given the exploratory nature of these analyses and the risk of false-positive findings associated with multiple comparisons, the following subgroup observations should be interpreted as hypothesis-generating rather than definitive conclusions, and findings from small-sample subgroups warrant particular caution. First, in the low and intermediate E2 groups, both BFR and EBR decreased with advancing age. This observation is consistent with multiple clinical and mechanistic studies, indicating that oocyte quality declines with age (32–36). However, this trend was not observed in the high E2 group. A possible explanation is that under high E2 conditions, the significantly increased oocyte yield may partially offset the decline in individual oocyte quality associated with advanced age, resulting in a higher probability of obtaining euploid embryos at the cohort level. Moreover, patients in the high E2 group typically represent those with good ovarian response, and this selection bias may further attenuate the negative impact of age on embryo quality. At the population level, in patients with preserved ovarian reserve, a greater number of retrieved oocytes is associated with a higher probability of obtaining euploid embryos. Additionally, we observed that extended stimulation duration and higher oocyte yield were associated with reduced BFR only in the low E2 group, but not in the intermediate or high E2 groups. This suggests that in patients with low E2 levels (indicative of poor ovarian response and/or diminished reserve), prolonged stimulation or excessive gonadotropin exposure may impair oocyte quality. However, these observations require validation in prospective studies specifically designed to address this question.

Some limitations of our study should be acknowledged. First, owing to the retrospective and single-center nature of the study, selection bias and residual confounding cannot be completely excluded, despite the large sample size and the application of multivariable adjustments. In particular, although we adjusted for key markers of ovarian reserve and response (AMH, AFC, number of oocytes retrieved, COS protocol, and gonadotropin dose), unmeasured or residual confounding related to ovarian responsiveness may persist, and the possibility that serum E2 serves as a surrogate rather than a causal factor for embryo euploidy cannot be ruled out. Furthermore, the primary association between high E2 and elevated EBR was modest in magnitude (aOR 1.295; 95% CI 1.028–1.631) with a borderline confidence interval, and thus should be interpreted as a weak observational association rather than a clinically meaningful protective effect. Second, analyses were restricted to BFR and EBR, without incorporating downstream clinical outcomes such as implantation, clinical pregnancy, or live birth. Consequently, the direct implications of elevated E2 levels on final reproductive outcomes remain to be elucidated. Additionally, as noted above, EBR represents a conditional measure restricted to biopsy-eligible blastocysts, and potential selection bias cannot be excluded. Third, other potentially relevant embryonic developmental parameters, including cleavage patterns, morphological characteristics, and omics data, were not assessed, thereby limiting proper elucidation of the mechanisms by which high E2 exerts its effects. Future multicenter prospective studies integrating multidimensional developmental and molecular indicators are needed to further validate the clinical significance of our findings and provide stronger evidence for individualized COS strategies.

## Conclusions

In summary, elevated hCG-trigger-day serum E2 was not significantly associated with blastocyst formation but was modestly associated with a higher euploid blastocyst rate in PGT cycles. These observational findings should be interpreted cautiously, as causality cannot be inferred and the association cannot be directly extrapolated to clinical pregnancy or live birth outcomes. Future prospective studies are warranted to corroborate these findings.

## Data Availability

The original contributions presented in the study are included in the article/**Supplementary Material**. Further inquiries can be directed to the corresponding authors.

## Supplementary material

Supplementary Figure 1 Restricted cubic spline analysis of the association between serum E2 on the hCG trigger day and BFR. Restricted cubic spline analysis with three knots (10th, 50th, 90th percentiles; 1693, 3868, 5000 pg/mL) was performed for BFR, adjusting for covariates identical to fully adjusted GEE Model II. Solid line: adjusted OR; shaded area: 95% CI; red dashed line: OR = 1. P-overall = 0.107; P-non-linear = 0.055.

Supplementary Figure 2 Restricted cubic spline analysis of the association between serum E2 on the hCG trigger day and EBR. Restricted cubic spline analysis with three knots (10th, 50th, 90th percentiles; 1693, 3868, 5000 pg/mL) was performed for EBR, adjusting for covariates identical to fully adjusted GEE Model II. Solid line: adjusted OR; shaded area: 95% CI; red dashed line: OR = 1. P-overall = 0.100; P-non-linear = 0.119.

Supplementary Table 1 Analysis of collinearity and interaction between total Gn dose and E2 level on the hCG trigger day.

Supplementary Table 2 Sensitivity analysis excluding number of oocytes retrieved from the fully adjusted GEE model II.
