# Supplementary material for: Association between serum estradiol levels on the hCG trigger day and euploid blastocyst rate in preimplantation genetic testing cycles: a retrospective cohort study
Source: Front Endocrinol (Lausanne). 2026 Oct 14;17:1935196. doi: 10.3389/fendo.2026.1935196 (PMC13713362; doi:10.3389/fendo.2026.1935196)
Supplement: Supplementary file 1 [file SupplementaryFile1.docx]

**Supplementary Figure 1.** Restricted cubic spline analysis of the association between serum E2 on the hCG trigger day and BFR.

Note: Restricted cubic spline analysis with three knots (10th, 50th, 90th percentiles; 1693, 3868, 5000 pg/mL) was performed for BFR, adjusting for covariates identical to fully adjusted GEE Model II. Solid line: adjusted OR; shaded area: 95% CI; red dashed line: OR = 1. P‑overall = 0.107; P‑non‑linear = 0.055.

**Supplementary Figure 2.** Restricted cubic spline analysis of the association between serum E2 on the hCG trigger day and EBR.

Note: Restricted cubic spline analysis with three knots (10th, 50th, 90th percentiles; 1693, 3868, 5000 pg/mL) was performed for EBR, adjusting for covariates identical to fully adjusted GEE Model II. Solid line: adjusted OR; shaded area: 95% CI; red dashed line: OR = 1. P‑overall = 0.100; P‑non‑linear = 0.119.

**Supplementary Table 1.** Analysis of Collinearity and Interaction Between Total Gn Dose and E2 Level on the hCG Trigger Day

| **Variable** | **Collinearity** | | **Interaction P value（BFR/EBR）** |
| --- | --- | --- | --- |
|  | **Tolerance** | **VIF** |  |
| **Continuous variables** |  |  | 0.159/0.701 |
| Total gonadotropin dose | 0.103 | 9.686 |  |
| Serum E2 level on hCG trigger day | 0.446 | 2.243 |  |
| **Categorical variables** |  |  | 0.908/0.927 |
| Total gonadotropin dose | 0.249 | 4.014 |  |
| Serum E2 level on hCG trigger day | 0.527 | 1.899 |  |

Note: Gn, gonadotropin; E2, estradiol; hCG, human chorionic gonadotropin; VIF, variance inflation factor. BFR, blastocyst formation rate; EBR, euploid blastocyst rate. Collinearity diagnostics for all covariates included in the fully adjusted GEE Model II confirmed that all VIF values were less than 6 (range: 1.137–5.399), indicating no significant multicollinearity in the final model. This table additionally presents pairwise collinearity statistics between total Gn dose and serum E2 (the clinically most relevant pair), as well as their interaction test results. Interaction P values were derived from interaction terms entered into the fully adjusted GEE Model II.

**Supplementary Table 2.** Sensitivity Analysis Excluding Number of Oocytes Retrieved from the Fully Adjusted GEE Model II

| **Model** | **BFR Group 3 vs. Group 1** |  | **EBR Group 3 vs. Group 1** |  |
| --- | --- | --- | --- | --- |
|  | aOR (95% CI) | P | aOR (95% CI) | P |
| Model II (with oocytes retrieved) | 1.050 (0.910–1.212) | 0.502 | 1.295 (1.028–1.631) | 0.028* |
| Model II (excluding oocytes retrieved) | 1.108 (0.973–1.261) | 0.122 | 1.232 (1.006–1.508) | 0.044* |

Note: aOR, adjusted odds ratio; CI, confidence interval; BFR, blastocyst formation rate; EBR, euploid blastocyst rate; GEE, generalized estimating equations. Both models were adjusted for all Model II covariates (female age, BMI, infertility type, PGT category, AMH, AFC, COS protocol, initial Gn dose, Gn duration, total Gn dose), except that the number of oocytes retrieved was excluded in the sensitivity model. Group 1 (low E2) served as the reference. *P < 0.05.
